# Correlation analysis of MR elastography and Ki-67 expression in intrahepatic cholangiocarcinoma

**DOI:** 10.1186/s13244-023-01559-7

**Published:** 2023-11-24

**Authors:** Shanshan Gao, Wei Sun, Yunfei Zhang, Feihang Wang, Kaipu Jin, Xianling Qian, Jing Han, Xiaolin Wang, Yongming Dai, Ruofan Sheng, Mengsu Zeng

**Affiliations:** 1 Department of Radiology, Zhongshan Hospital, Fudan University, Shanghai, 200032 China; 2 Shanghai Institute of Medical Imaging, Shanghai, 200032 China; 3 Central Research Institute, United Imaging Healthcare, Shanghai, 201800 China; 4 Department of Interventional Radiology, Zhongshan Hospital, Fudan University, Shanghai, 200032 China; 5 Department of Pathology, Zhongshan Hospital, Fudan University, Shanghai, 200032 China; 6 Department of Radiology, Zhongshan Hospital (Xiamen), Fudan University, Fujian, 361006 China; 7 Department of Cancer Center, Zhongshan Hospital, Fudan University, Shanghai, 200032 China

**Keywords:** Intrahepatic cholangiocarcinoma, Magnetic resonance elastography, Ki-67

## Abstract

**Background:**

Intrahepatic cholangiocarcinoma (iCCA) is an aggressive primary liver cancer with dismal outcome, high Ki-67 expression is associated with active progression and poor prognosis of iCCA, the application of MRE in the prediction of iCCA Ki-67 expression has not yet been investigated until now. We aimed to evaluate the value of magnetic resonance elastography (MRE) in assessing Ki-67 expression for iCCA.

**Results:**

In the whole cohort, 97 patients (57 high Ki-67 and 40 low Ki-67; 58 males, 39 females; mean age, 58.89 years, ranges 36–70 years) were included. At the multivariate analysis, tumor stiffness (odds ratio (OR) = 1.669 [95% CI: 1.307–2.131], *p* < 0.001) and tumor apparent diffusion coefficient (ADC) (OR = 0.030 [95% CI: 0.002, 0.476], *p* = 0.013) were independent significant variables associated with Ki-67. Areas under the curve of tumor stiffness for the identification of high Ki-67 were 0.796 (95% CI 0.702, 0.871). Tumor stiffness was moderately correlated with Ki-67 level (*r* = 0.593, *p* < 0.001). When both predictive variables of tumor stiffness and ADC were integrated, the best performance was achieved with area under the curve values of 0.864 (95% CI 0.780–0.926).

**Conclusion:**

MRE-based tumor stiffness correlated with Ki-67 in iCCA and could be investigated as a potential prognostic biomarker. The combined model incorporating both tumor stiffness and ADC increased the predictive performance.

**Critical relevance statement:**

MRE-based tumor stiffness might be a surrogate imaging biomarker to predict Ki-67 expression in intrahepatic cholangiocarcinoma patients, reflecting tumor cellular proliferation. The combined model incorporating both tumor stiffness and apparent diffusion coefficient increased the predictive performance.

**Key points:**

• MRE-based tumor stiffness shows a significant correlation with Ki-67.

• The combined model incorporating tumor stiffness and apparent diffusion coefficient demonstrated an optimized predictive performance for Ki-67 expression.

• MRE-based tumor stiffness could be investigated as a potential prognostic biomarker for intrahepatic cholangiocarcinoma.

**Graphical Abstract:**

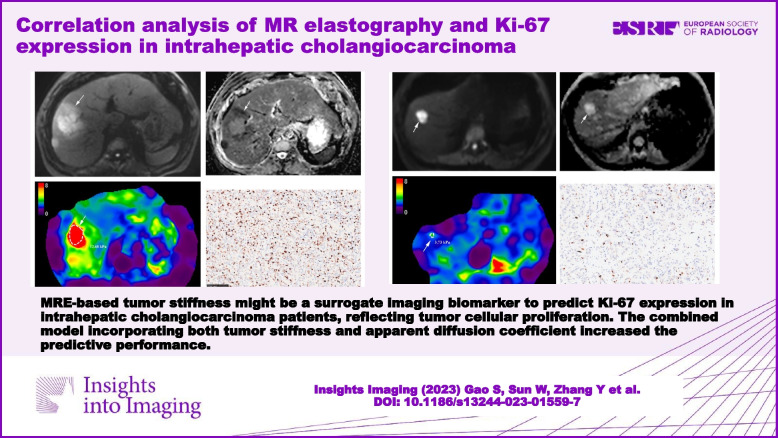

**Supplementary Information:**

The online version contains supplementary material available at 10.1186/s13244-023-01559-7.

## Introduction

Intrahepatic cholangiocarcinoma (iCCA) is the second most common primary liver cancer after hepatocellular carcinoma with an increasing incidence worldwide [1–3]. Surgical resection of the primary tumor site offers the best chance to prolong the survival time of patients [4]. Unfortunately, because of its high recurrence and metastasis rates, the five-year survival rate after surgery is only 20–40% [5–7]. Ki-67 is a nuclear antigen associated with cell proliferation, which is an important biomarker for biological behaviors relating to the development, progression, and metastasis in various malignant tumors [8–10]. High Ki-67 level is reported to be correlated with poor survival in cholangiocarcinoma, which can be used to evaluate the biological behavior and prognosis in iCCA [11–14]. Hence, Ki-67 may be a valuable biomarker for prognosis prediction in iCCA. Until now, liver biopsy is necessary for the preoperative measurement of Ki-67. But as an invasive procedure, biopsy may lead to potential complications, and its results are often inconsistent with post-operative pathology because of the heterogeneity of tumors and limited sampling. For these reasons, it is crucial to search for accurate, minimally invasive, and even non-invasive methods to predict the Ki-67 expression before surgery.

Due to the diverse information provided and the emerging functional imaging technique, MRI is considered to be the preferred imaging means for the diagnosis and assessment of liver lesions [15]. However, it is difficult to determine the level of Ki-67 by conventional techniques [11, 16]. Magnetic resonance elastography (MRE) is an emerging functional technique capable of quantifying tissue mechanical properties in vivo [17, 18], which shows acknowledged diagnostic performance in staging liver fibrosis [19]. Nowadays, it has been more and more widely applied in the characterization, treatment monitoring, and prognosis evaluation of liver cancers [18, 20]. However, the application of MRE in the prediction of iCCA Ki-67 expression has not yet been investigated until now.

Therefore, the purpose of this study was to evaluate the correlation of MRE-based tumor stiffness and Ki-67 expression in iCCA.

## Materials and methods

### Participants

**Fig. 1:**
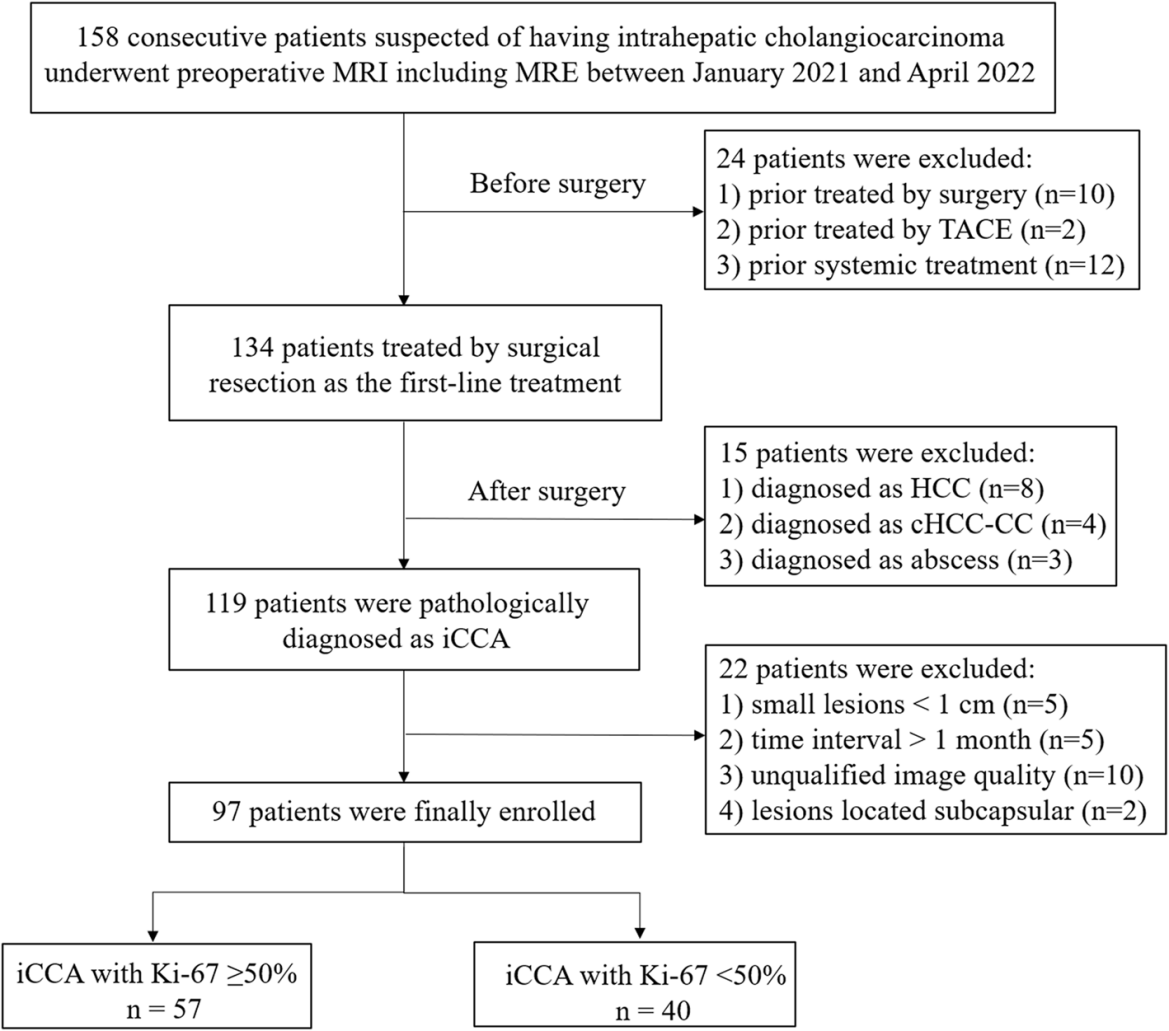
Flowchart of the inclusion and exclusion criteria. MRE, magnetic resonance elastography; TACE, transcatheter arterial chemoembolization; HCC, hepatocellular carcinoma; cHCC-CC, combined hepatocellular carcinoma-cholangiocarcinoma; iCCA, intrahepatic cholangiocarcinoma

This study prospectively enrolled consecutive adult participants who were suspected of having iCCA based on previous CT or ultrasonography examinations and were referred to our hospital for liver surgery between January 2021 and April 2022. The study was approved by the institutional review board and written informed consent was obtained from each participant. Participants with no contraindications for gadopentetate dimeglumine underwent contrast-enhanced MRI examination with MRE sequence in our institute, all patients should fast 4–6 h prior to the examination. All patients included underwent liver surgery. Exclusion criteria were (1) previous history of local or systemic oncologic treatment; (2) lesions pathologically diagnosed as other tumors rather than iCCA; (3) small tumors less than 1 cm (to avoid incorrect tumor stiffness measurements); (4) lesions located subcapsular (areas within 1 cm from the liver capsule); (5) time interval between MR scan and surgery more than 1 month; and (6) difficult to measure stiffness values because of poor image quality. The flowchart of the inclusion and exclusion criteria is presented in Fig. 1.

### Image acquisition

The two-dimensional MRE examinations were acquired with a standard commercial equipment at a 3.0-T MRI scanner (uMR 770, United Imaging Healthcare, Shanghai, China). The shear wave frequency was set as 60 Hz. A passive pneumatic driver located on the right lobe of the liver and centered at the level of the xiphisternum was utilized to generate mechanical vibrations and produce propagating shear waves in the imaging region. The 2D-MRE scanning protocol was based on an axial spin-echo echo-planner-imaging sequence and the detailed scanning parameters are as follows: TR: 1000.2 ms, TE: 44.6 ms, flip angle: 90°, field of view: 420 × 420 mm^2^, slice thickness: 8 mm, matrix: 256 × 256, bandwidth: 1500 Hz, scanning time: 10 s. MRE sequence was performed during one breath-hold at end-expiration. The magnitude, phase, wave, and elastographic images were transferred offline and the parametric maps were generated with a custom software package (MRE Quant, Mayo Clinic, Rochester, MN).

Other conventional sequences consisted of a breath-hold T2-weighted fat-suppressed fast spin-echo sequence, T1-weighted in-phase, and opposed-phase gradient echo sequence, respiratory-triggering single-shot spin-echo echoplanar diffusion-weighted imaging (DWI) with *b* values of 0, 50, and 500 s/mm^2^. Dynamic imaging was performed with a 3D breath-hold T1-weighted fat-suppressed gradient-echo sequence, prior to and after intravenous administration of gadopentetate dimeglumine (Magnevist; Bayer HealthCare, Berlin, Germany) at a rate of 2 mL/s and at a dose of 0.1 mmol/kg. The arterial phase acquisitions were automatically triggered when contrast media reached the ascending aorta. Subsequent acquisitions were performed at 60–70 s for the portal venous phase and 180 s for the delay phase. Detailed parameters are shown in Table S1.

### Tumor and liver stiffness measurement

MRE images were independently evaluated by two radiologists (with 5 and 10 years of experience in liver MRI, respectively). The reviewers were aware that the patients had iCCA, but were blinded to all other information, including the Ki-67 labeling index, clinical history, and laboratory results. Regions of interest (ROIs) were drawn for the lesion and unaffected hepatic parenchyma at the same level and segment, the tumors were guaranteed to be located in their entirety in the 95% confidence area of the map. By using T2-weighted and contrast-enhanced images as references, ROIs were manually drawn on magnitude images, including the solid tumor area as large as possible, and then copied to the stiffness maps, the most peripheral portions of tumors were avoided to exclude partial volume effects. Great care was also taken to avoid areas of significant wave interference and necrosis. For each case, 3 adjacent slices with a maximum cross-section of the tumor were chosen. Stiffness values of the lesion and liver were measured on each slice, and the average value of the 3 measurements was used. Mean values measured by two observers were averaged for final analysis.

### Conventional MRI features

The conventional MRI images were assessed by another two radiologists (with 7 and 13 years of experience in liver MRI, respectively), who were blinded to the results of the Ki-67 labeling index and MRE analysis. When disagreement occurred in the qualitative analysis between the two observers, a consensus review was made by a third senior radiologist (with 35 years of experience in abdominal MRI) for the final decision.

The qualitative imaging parameters were evaluated as follows: (a) location (left/right/left and right/caudal lobe); (b) tumor margin (well-defined: well-defined tumor with distinct contour/ill-defined: ill-defined tumor with indistinct contour); (c) signal homogeneity (homogeneous: the entire tumor was uniform with homogeneous signal inside/heterogeneous: the entire tumor was nonuniform with heterogeneous portion compared with the main body of tumor). Signal homogeneity was evaluated on T2-weighted imaging, a cut-off of ≥ 10% heterogeneous regions of the entire tumor was regarded as heterogeneous, and heterogeneous regions < 10% was defined as homogeneous; (d) arterial phase enhancement (global hyperenhancement: increased signal relative to the liver parenchyma, involving the totality of lesion/partial hyperenhancement: increased signal involving ≥ 25% of the lesion, except the central area/peripheral enhancement: increased signal limited to the periphery of the lesion, involving < 25% of the lesion); (e) enhancement pattern (progressive: increasing enhancement over time/persistent: invariable enhancement over time/degressive-washout: decreasing enhancement over time); (f) arterial peritumoral hyperenhancement (defined as fuzzy-marginated hyperenhancement outside the tumor borders that becomes isointense with normal liver parenchyma in later dynamic phases); (g) enhancing tumor capsule (smooth, uniform, sharp border around most or all of tumor, and visible as an enhancing rim in portal venous or delayed phases); (h) targetoid appearance (rim arterial phase hyperenhancement, peripheral washout, delayed central enhancement, or targetoid restriction on DWI); (i) bile duct dilation with diameter ≥ 5mm; (j) liver capsule retraction; (k) hemorrhage in mass (defined as high-signal foci on T1-weighted images with variable signal intensity on T2-weighted images); (l) necrotic or cystic portion in mass (defined as bright signal foci on T2-weighted images without contrast enhancement); (m) central scar (central or eccentric area within a tumor with stellate appearance and radiating septa); (n) central darkness on T2-weighted imaging (central signal darker than liver signal); (o) vessel invasion (defined when vessels cannot be separated from the mass, with the rough change of the wall or narrowing and occlusion of the lumen); (p) lymphadenectasis; (q) distant metastasis. In cases of multiple tumors with satellite nodules, the major tumor with the largest size was analyzed.

Tumor apparent diffusion coefficient (ADC) values were measured by ROIs manually drawn in ADC maps. Slice locations of ROIs were selected in consistent with stiffness measurement as much as possible, avoiding large vessels, necrosis, hemorrhage, and artifacts. Similarly, 3 ROIs were drawn for each case and the average value was used. Tumor size (the largest diameter) was measured in the delay phase by the senior reviewer.

### Histopathological evaluation

Pathologic characteristics were evaluated by an experienced pathologist with 30 years of experience in liver pathology. The Ki-67 level was determined by using the percentiles of immunoreactive cells from 1000 malignant cells (× 400), and scoring was performed in the areas with the highest number of positive nuclei (hot spot) within the tumor. Then, we classified iCCAs into the “high Ki-67 group” (positive staining ratio ≥ 50%) and “low Ki-67 group” (positive staining ratio < 50%), referring to prior researchers [14, 21, 22].

### Statistical analysis

Statistical analysis was performed using SPSS 26.0 (SPSS, Armonk, NY, USA) and MedCalc software (www.medcalc.org). Continuous variables were compared with the Student’s *t*-test or Mann–Whitney *U*-test; categorical variables were compared using Pearson’s chi-squared test or Fisher’s exact test. The interobserver agreement on qualitative imaging findings was determined using kappa statistics: poor, 0–0.2; fair, 0.2–0.4; moderate, 0.4–0.6; good, 0.6–0.8; and excellent, 0.8–1.0. The interobserver agreement on the quantitative findings was determined using intraclass correlation coefficient (ICC) (two-way random, absolute agreement, single measurements): poor, < 0.5; moderate, 0.5–0.75; good, 0.75–0.9; and excellent, > 0.9. Variables showing *p* < 0.05 in the univariate logistic regression analysis were applied to multivariate logistic regression analysis. Receiver operating characteristic analysis was performed to assess the ability of tumor stiffness in distinguishing the high Ki-67 iCCAs from the low Ki-67 iCCAs, and the specificity, sensitivity, and accuracy were calculated for the corresponding area under the curve (AUC). In addition, the threshold values for tumor stiffness and ADC were evaluated based on the best Youden’s index on the receiving operating characteristic curve. Spearman correlation analysis was performed to analyze the correlations between tumor stiffness and the Ki-67 labeling index. The correlation was very weak for absolute value of correlation coefficient |*r*|= 0.0–0.2, weak for |*r*|= 0.2–0.4, moderate for |*r*|= 0.4–0.7, strong for |*r*|= 0.7–0.9, very strong for |*r*|= 0.9–1.0.

## Results

### Clinical characteristics

Baseline patients’ demographic characteristics related to Ki-67 are demonstrated in Table 1. Totally 97 patients (57 high Ki-67 and 40 low Ki-67; 58 males, 39 females; mean age, 58.89 years, ranges 36–70 years) were included. In our cohort, the sex (*p* = 0.002), serum carbohydrate antigen19-9 level (*p* = 0.015) were different between the high Ki-67 and low Ki-67 groups.

**Table 1:** Baseline clinical characteristics of the patient

Variable		**High Ki-67** (***n*** = 57)	**Low Ki-67** (***n*** = 40)	***p***
Age (years)^a^	62 (52, 66)	63 (51.5, 67)	59.5 (54, 66)	0.615
Sex, male/female	57 (58.8%)/40 (41.2%)	41 (71.9%)/16 (28.1%)	16 (40.0%)/24 (60.0%)	0.002*
AFP ≥ 20/ < 20 ng/mL	9 (9.3%)/88 (90.7%)	6 (10.5%)/51 (89.5%)	3 (7.5%)/37 (92.5%)	0.732
CEA ≥ 5/ < 5 ng/mL	29 (29.9%)/68 (70.1%)	21 (36.8%)/36 (63.2%)	8 (20.0%)/32 (80.0%)	0.074
CA19-9 ≥ 37/ < 37 ng/mL	53 (54.6%)/44 (45.4%)	37 (64.9%)/20 (35.1%)	16 (40.0%)/24 (60.0%)	0.015*
TBil > 20.4/ ≤ 20.4 μmol/L	5 (5.2%)/92 (94.8%)	1 (1.8%)/56 (98.2%)	4 (10.0%)/36 (90.0%)	0.156
ALT > 35/ ≤ 35 U/L	20 (20.6%)/77 (79.4%)	11 (19.3%)/46 (80.7%)	9 (22.5%)/31 (77.5%)	0.701
AST > 40/ ≤ 40 U/L	16 (16.5%)/81 (83.5%)	8 (14.0%)/49 (86.0%)	8 (20.0%)/32 (80.0%)	0.436
ALP > 125/ ≤ 125 U/L	33 (34.0%)/64 (66.0%)	22 (38.6%)/35 (61.4%)	11 (27.5%)/29 (72.5%)	0.256
γGGT > 60/ ≤ 60 U/L	53 (54.6%)/44 (45.4%)	35 (61.4%)/22 (38.6%)	18 (45.0%)/22 (55.0%)	0.110
Chronic liver disease Y/N	29 (29.9%)/68 (70.1%)	17 (29.8%)/40 (70.2%)	12 (30.0%)/28 (70.0%)	0.777
Hepatitis B	26 (89.7%)	16 (94.1%)	10 (83.3%)	
Hepatitis C	0 (0.0%)	0 (0.0%)	0 (0.0%)	
Hepatitis B + C	1 (3.4%)	0 (0.0%)	1 (8.3%)	
Other causes	2 (6.9%)	1 (5.9%)	1 (8.3%)	
Cirrhosis Y/N	22 (22.7%)/75 (77.3%)	12 (21.1%)/45 (78.9%)	10 (25.0%)/30 (75.0%)	0.648

Unless otherwise indicated, data are numbers of patients

Quantitative variables are analyzed using the Mann–Whitney *U*-test; categorical variables are analyzed using Pearson’s *χ*^*2*^ test or Fisher’s exact test, as appropriate

*AFP* alpha fetoprotein, *CEA* carcinoembryonic antigen, *CA19-9* cancer antigen 19–9, *TBil* total bilirubin, *ALT* alanine aminotransferase, *AST* aspartate aminotransaminase, *ALP* alkaline phosphatase, *γGGT* γ-glutamyltransferase

^a^Data are median (Q1, Q3)

^*^Data are statistically significant results

### MRE stiffness measurements

**Fig. 2:**
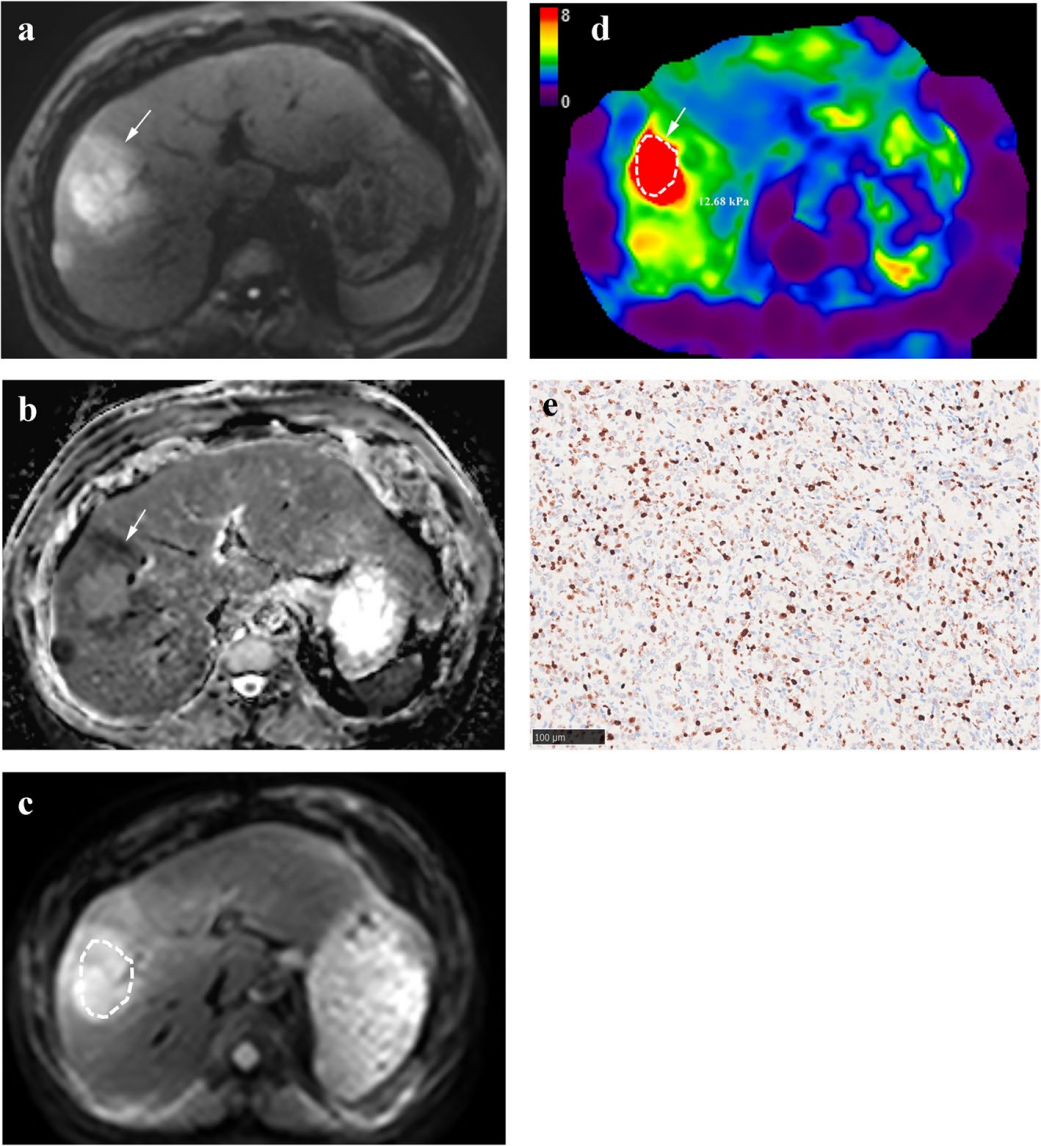
Intrahepatic cholangiocarcinoma of the high Ki-67 group in a 62-year-old man (arrows) showed (**a**) hyperintensity on diffusion-weighted image (*b* = 500 s/mm^2^), (**b**) hypointensity in the tumor periphery on apparent diffusion coefficient map (1.054 × 10^3^ mm^2^/s). **c** The magnitude image and (**d**) stiffness map showed a high tumor stiffness value of 12.68 kPa. **e** Photomicrograph (Ki-67 immunostaining × 200) showed Ki-67 labeling index of 60%. A small satellite nodule can be seen beside the major lesion

**Fig. 3:**
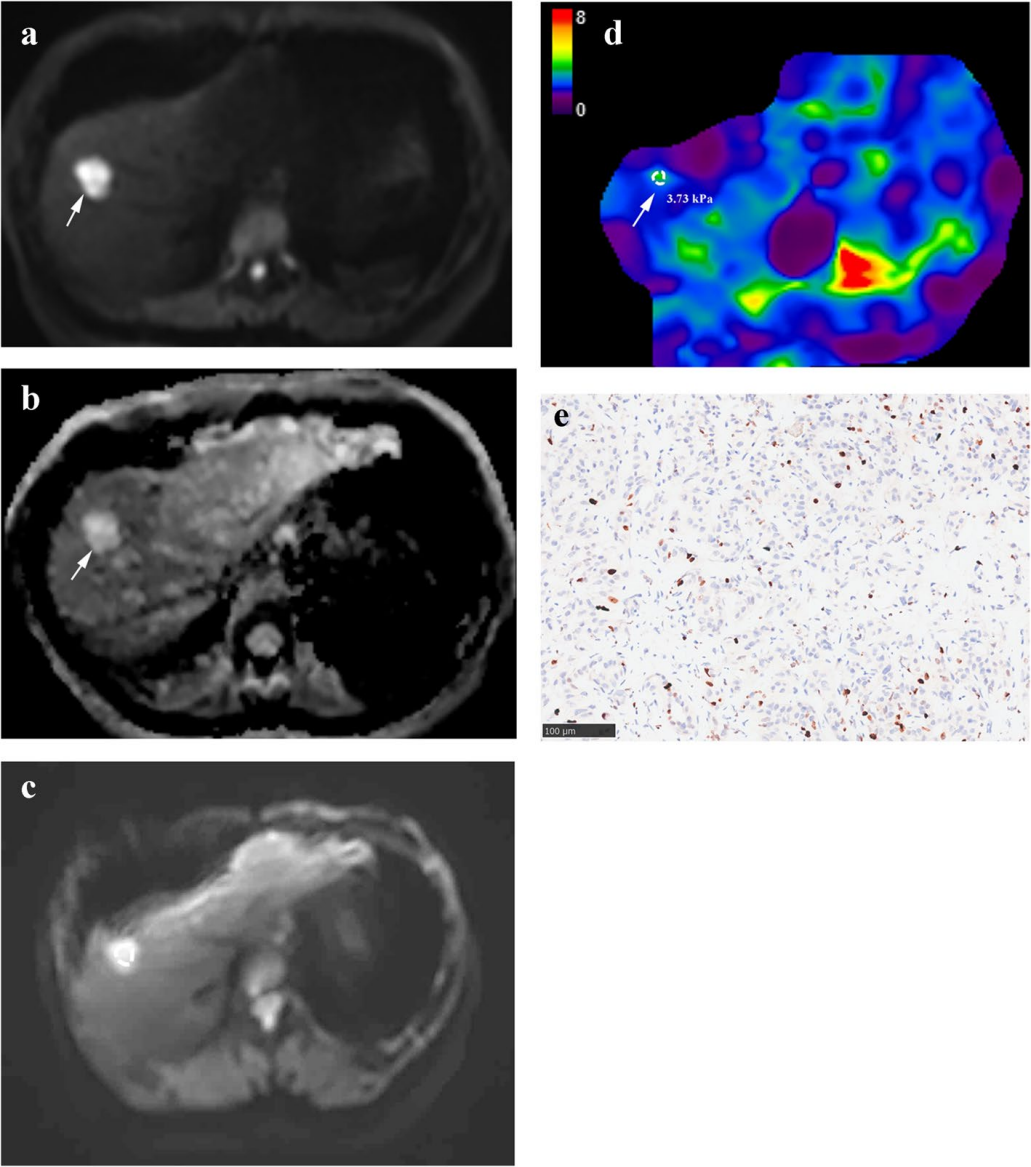
Intrahepatic cholangiocarcinoma of the low Ki-67 group in a 51-year-old female (arrows) showed (**a**) hyperintensity on DWI (*b* = 500 s/mm^2^); (**b**) hyperintensity on apparent diffusion coefficient map (2.390 × 10^3^ mm^2^/s). **c** The magnitude image and (**d**) stiffness map showed a low tumor stiffness value of 3.73 kPa. **e** Photomicrograph (Ki-67 immunostaining × 200) showed Ki-67 labeling index of 10%

The tumor stiffness for all iCCAs in the whole cohort was median 7.94 kPa, ranges 14.3 kPa-3.73 kPa, Q1-Q3 6.23, 7.94 and 11.09 kPa. The mean values of MRE stiffness measured by the two observers in the two groups are displayed in Table 2. Tumor stiffness of the two observers was significantly higher in the high Ki-67 group (Fig. 2) than in the low Ki-67 group (Fig. 3) (*p* < 0.001). Liver stiffness values were not significantly different between groups (*p* = 0.936 and 0.947). The interobserver agreements were excellent for both tumor stiffness (ICC = 0.863 [95% confidence interval (CI): 0.802, 0.906]) and liver stiffness (ICC = 0.898 [95% CI: 0.852, 0.931]) measurements.

**Table 2:** Stiffness values of the high Ki-67 and low Ki-67 groups for each observer in the whole cohort

Parameters	Observer 1		*p*	Observer 2		*p*
	High Ki-67	Low Ki-67		High Ki-67	Low Ki-67	
Tumor stiffness (kPa)	10.68 (6.83, 12.75)	6.78 (5.77, 8.61)	< 0.001^*^	9.81 (7.11, 12.01)	6.36 (5.66, 7.96)	< 0.001^*^
Liver stiffness (kPa)	2.73 (2.33, 3.58)	2.75 (2.26, 3.54)	0.936	2.73 (2.25, 3.69)	2.76 (2.24, 3.67)	0.947

Data are median (Q1, Q3)

^*^p < 0.05

### MR imaging characteristics related to Ki-67

The imaging characteristics of tumors related to Ki-67 are presented in Table 3. In our cohort, targetoid appearance (*χ*^*2*^ = 7.153, *p* = 0.007), tumor ADC (*p* < 0.001), and tumor stiffness (*p* < 0.001) were significantly different between the high Ki-67 and low Ki-67 iCCAs. Interobserver agreements of the imaging findings were good to excellent for all parameters (kappa = 0.731–0.935 for qualitative imaging features (Table S2), and ICC = 0.913 [95% CI: 0.872, 0.941] for ADC measurements). According to the ROC analysis, the cutoff value for tumor stiffness was set as 8.90 kPa, and the cutoff value for ADC was 1.03 × 10^3^ mm^2^/s.

**Table 3:** MR imaging characteristics of tumors

Variable		**High Ki-67 (*n* = 57)**	**Low Ki-67 (*n* = 40)**	***p***
Largest diameter (cm)^a^	5.74 ± 2.37	5.61 ± 2.16	5.91 ± 2.65	0.544
Location left/right/left + right/caudal lobe	43 (44.3%)/45 (46.4%)/7 (7.2%)/2 (2.1%)	26 (45.5%)/27 (47.4%)/3 (5.3%)/1 (1.8%)	17 (42.5%)/18 (45%)/4 (10.0%)/1 (2.5%)	0.830
Tumor margin Well-defined/ill-defined	69 (71.1%)/28 (28.9%)	42 (73.7%)/15 (26.3%)	27 (67.5%)/13 (32.5%)	0.508
Signal homogeneity Homogeneous/heterogeneous	24 (24.7%)/73 (75.3%)	14 (24.6%)/43 (75.4%)	10 (25.0%)/30 (75.0%)	0.961
Arterial enhancement Global/partial/peripheral enhancement	5 (5.2%)/15 (15.5%)/77 (79.4%)	1 (1.8%)/6 (10.5%)/50 (87.7%)	4 (10.0%)/9 (22.5%)/27 (67.5%)	0.036*
Enhancement pattern Progressive/persistent/degressive-washout	84 (86.6%)/4 (4.1%)/9 (9.3%)	51 (89.5%)/3 (5.3%)/3 (5.3%)	33 (82.5%)/1 (2.5%)/6 (15.0%)	0.295
APHE Y/N	75 (77.3%)/22 (22.7%)	45 (78.9%)/12 (21.1%)	30 (75.0%)/10 (25.0%)	0.648
Capsule Y/N	20 (20.6%)/77 (79.4%)	15 (26.3%)/42 (73.7%)	5 (12.5%)/35 (87.5%)	0.098
Targetoid appearance Y/N	76 (78.4%)/21 (21.6%)	50 (87.7%)/7 (12.3%)	26 (65.0%)/14 (35.0%)	0.007*
Satellite nodules Y/N	29 (29.9%)/68 (70.1%)	16(28.1%)/41 (71.9%)	13 (32.5%)/27 (67.5%)	0.639
Bile duct dilation Y/N	61 (62.9%)/36 (37.1%)	34 (59.6%)/23 (40.4%)	27 (67.5%)/13 (32.5%)	0.431
Liver capsule retraction Y/N	37 (38.1%)/60 (61.9%)	22 (38.6%)/35 (61.4%)	15 (37.5%)/25 (62.5%)	0.913
Hemorrhage in mass Y/N	4 (4.1%)/93 (95.9%)	2 (3.5%)/55 (96.5%)	2(5.0%)/38 (95.0%)	1.000
Necrotic or cystic portion Y/N	36 (37.1%)/61 (62.9%)	22 (38.6%)/35 (61.4%)	14 (35.0%)/26 (65.0%)	0.718
Central scar Y/N	48 (49.5%)/49 (50.5%)	29 (50.9%)/28 (49.1%)	19 (47.5%)/21 (52.5%)	0.743
Central darkness on T2WI Y/N	45 (46.4%)/52 (53.6%)	27(47.4%)/30 (52.6%)	18 (45.0%)/22 (55.0%)	0.818
Vessel invasion Y/N	76 (78.4%)/21 (21.6%)	46 (80.7%)/11 (19.3%)	30 (75.0%)10/ (25.0%)	0.502
Lymphadenectasis Y/N	42 (43.3%)/55 (56.7%)	23 (40.4%)/34 (59.6%)	19 (47.5%)/21 (52.5%)	0.484
Distant metastasis Y/N	11 (11.3%)/86 (88.7%)	8 (14.0%)/49 (86.0%)	3 (7.5%)/37 (92.5%)	0.517
Tumor ADC (× 10^3^ mm^2^/s)^b^	1.13 (0.96, 1.30)	1.02 (0.87, 1.22)	1.19 (1.06, 1.42)	< 0.001*
Tumor stiffness (kPa)^b^	7.94 (6.23, 11.09)	10.16 (7.17, 12.04)	6.37 (5.65, 7.69)	< 0.001*
Liver stiffness (kPa)^b^	2.74 (2.30, 3.52)	2.65 (2.30, 3.64)	2.77 (2.31, 3.47)	0.959

Unless otherwise specified, data are numbers of lesions

Quantitative variables are analyzed using the independent *t*-test or Mann–Whitney *U*-test; categorical variables are analyzed using Pearson’s *χ*^*2*^ test or Fisher’s exact test, as appropriate

*APHE* arterial peritumoral hyperenhancement, *T2WI* T2-weighted imaging, *ADC* apparent diffusion coefficient

^a^Data are mean ± standard deviation

^b^Data are median (Q1, Q3)

^*^ *p* < 0.05

The results of the univariate and multivariate analyses of features related to Ki-67 are described in Table 4. At the multivariate analysis, tumor stiffness (odds ratio (OR) = 1.669 [95% CI: 1.307–2.131], *p* < 0.001) and tumor ADC (OR = 0.030 [95% CI: 0.002, 0.476], *p* = 0.013) were independent significant variables associated with Ki-67.

**Table 4:** Univariate and multivariate analyses of risk factors for Ki-67 level in the training cohort

Variables	Univariate analysis		Multivariate analysis	
	Odds ratio	*p*	Odds ratio	*p*
Sex	3.844 (1.632–9.053)	0.002*	…	…
CA19-9	2.775 (1.205–6.391)	0.016*	…	…
Largest diameter	0.948 (0.798–1.126)	0.540	…	…
Tumor margin	1.348 (0.556–3.270)	0.509	…	…
Signal homogeneity	1.024 (0.402–2.610)	0.961	…	…
Arterial enhancement	…	…	…	…
Global^a^	…	…	…	…
Partial	2.667 (0.237–30.066)	0.427	…	…
Peripheral enhancement	7.407 (0.788–69.632)	0.08	…	…
Enhancement pattern	…	…	…	…
Progressive^a^	…	…	…	…
Persistent	1.941 (0.194–19.461)	0.573	…	…
Degressive-washout	0.324 (0.076–1.384)	0.128	…	…
APHE	1.250 (0.480–3.258)	0.648	…	…
Capsule	2.500 (0.826–7.564)	0.105	…	…
Targetoid appearance	3.846 (1.382–10.705)	0.010*	…	…
Satellite nodules	1.234 (0.513–2.970)	0.639	…	…
Bile duct dilation	1.405 (0.602–3.278)	0.432	…	…
Liver capsule retraction	1.048 (0.455–2.410)	0.913	…	…
Hemorrhage in mass	1.447 (0.195–10.728)	0.718	…	…
Necrotic or cystic portion	1.167 (0.504–2.705)	0.718	…	…
Central scar	1.145 (0.510–2.571)	0.743	…	…
Central darkness on T2WI	1.100 (0.489–2.476)	0.818	…	…
Vessel invasion	1.394 (0.527–3.685)	0.503	…	…
Lymphadenectasis	1.337 (0.592–3.023)	0.485	…	…
Tumor ADC	0.028 (0.004–0.208)	< 0.001*	0.030 (0.002–0.476)	0.013
Tumor stiffness	1.640 (1.327–2.027)	< 0.001*	1.669 (1.307–2.131)	< 0.001*
Liver stiffness	0.950 (0.694–1.299)	0.747	…	…

*CEA* carcinoembryonic antigen, *CA19-9* cancer antigen 19–9, *APHE* arterial peritumoral hyperenhancement, *T2WI* T2-weighted imaging, *ADC* apparent diffusion coefficient, *OR* odds ratio, *95% CI* 95% confidence interval

… indicate variables not included in the equation of the multivariate logistic stepwise regression model

^a^Data were used as the reference category

^*^p < 0.05

### Diagnostic performance of tumor stiffness and ADC related to Ki-67

Diagnostic characteristics of the significant features (tumor stiffness and ADC) and their combination for predicting Ki-67 level are demonstrated in Table 5. In our study, the AUC of tumor stiffness for identifying the high Ki-67 group was 0.796 (95% CI 0.702, 0.871), with a sensitivity of 68.42%, specificity of 92.50%, and accuracy of 78.35%. Tumor stiffness was moderately correlated with the Ki-67 labeling index (*r* = 0.593, *p* < 0.001). While liver stiffness showed no significant correlations with Ki-67 in our cohorts (*p* > 0.05). There was no correlation between tumor stiffness and ADC (*r* =  − 0.196, *p* = 0.055).

**Table 5:** Diagnostic performance of the significant findings and their combination for predicting Ki-67 of intrahepatic cholangiocarcinoma

Variable	**AUC**	**Sensitivity**	**Specificity**	**Accuracy**
Tumor ADC	0.718	50.88%	85.00%	64.95%
Tumor stiffness	0.796	68.42%	92.50%	78.35%
Combined model	0.864	78.95%	82.50%	78.35%

*AUC* area under the curve, *ADC* apparent diffusion coefficient

When both significant features of tumor stiffness and ADC were combined, corresponding AUC values were 0.864 (95% CI 0.780–0.926). The combined model achieved the best diagnostic performance compared to the individual imaging features of tumor stiffness (*z* = 2.539, *p* = 0.0111) or ADC (*z* = 2.986, *p* = 0.0028) alone.

## Discussion

In the present study, we found that MRE-based tumor stiffness showed a significant correlation with Ki-67. Furthermore, increased tumor stiffness can be a reliable predictor for high Ki-67 iCCA as an independent variable, achieving satisfactory diagnostic performance in cohorts. Hu et al. have proven that MRE-based c and φ-maps can be served as important parameters to assess tumor proliferation status in hepatocellular carcinomas [23]. But to the best of our knowledge, it is the first time to explore the association between tumor stiffness and Ki-67 in iCCA patients.

According to our results, tumor stiffness was an independent predictor for Ki-67 in iCCA. Increased tumor stiffness was reported to be related to poor differentiation, high recurrence rate, and unfavorable survival, indicating it as a potential biomarker reflecting the aggressiveness of tumors [20, 24–26]. This is in accordance with our results that cases with higher levels of Ki-67 displayed increased tumor stiffness. We considered that this may have guiding significance for the selection of the puncture area for tumor biopsy, and the area with the highest tumor hardness should be selected as much as possible. Tumor stiffness reflects the inherent mechanical properties of lesions and is closely related to various constitutes of microstructures, responding to underlying tumor heterogeneity [17, 27]. While the increased stiffness and cellular density might be correlated as well with the well-known desmoplastic reaction of cholangiocarcinoma, another component associated with biological aggressiveness and poor prognosis [28]. The MRE stiffness may not only demonstrate the cellular proliferation, but also the desmoplastic response induced by fibroblasts and extracellular collagen matrix. So elastometric features might be a surrogate biomarker of cellular proliferation. What’s more, it can also provide more information for clinicians to select further treatment.

We also identified the role of ADC in predicting Ki-67 of iCCA, consistent with previous study [29]. Diffusion-weighted imaging provides insights into the microstructure of tissues by reflecting the micro-diffusivity of water molecules. iCCA with a high level of Ki-67 had a lower ADC value, the possible mechanism may be that high cell proliferation activity is associated with decreased diffusivity, resulting in lower ADC values [11].

Accordingly, MRE showed promising diagnostic performance, displaying considerable efficacy in preoperative prediction of Ki-67 expression in iCCA. Moreover, the stiffness measurement displayed excellent interobserver agreement with no need of contrast media employment, which may be a convenient, noninvasive, and repeatable imaging biomarker for preoperative assessment of tumor proliferation in iCCA. In our study, we further incorporated tumor stiffness and the conventional imaging feature of tumor ADC to build a combined diagnostic model, and it demonstrated an optimized predictive performance with the largest AUC, which may provide a promising and reliable tool to assist in treatment decision-making and outcome prediction for iCCA patients in clinical work.

There were several shortcomings to be acknowledged. First, this was a single-center study with a relatively small sample size, which may lead to biased results. Therefore, the value of MRE in the prediction of Ki-67 requires to be validated in a multicenter study with a larger sample size and external validation. Second, few cases may have poor image quality due to severe artifacts, but these patients were excluded from our study according to the exclusion criteria. Third, we defined the expression of Ki-67 ≥ 50% as the high Ki-67 group according to previous studies [14, 21, 22]. However, several studies chose different cutoff values [30, 31]. Fourth, conventional extracellular contrast-enhanced MRI was performed in this study, as Gadoxetic acid was not routinely applied and was not covered by health insurance in our country. Further studies using Gadoxetic acid with analysis of hepatobiliary-phase features are warranted. Fifth, point-to-point correlation analysis for tumor stiffness and Ki-67 expression was not analyzed and needs to be further investigated in our future work. Finally, 2D-MRE can only provide 3 scanning layers for limited ROI selection, whole-tumor evaluation could not be investigated.

In conclusion, as a noninvasive imaging tool, MRE-based tumor stiffness correlated with Ki-67 in iCCA and could be investigated as a potential prognostic biomarker in future. Furthermore, the combined model incorporating both tumor stiffness and the conventional MRI feature of tumor ADC increased the predictive performance for Ki-67.

### Supplementary Information

**Additional file 1: Table S1.** Sequence parameters. **Table S2.** The intraclass correlation coefficient between two observers in the whole cohort for the qualitative MRI features.

## Data Availability

Data presented in this study are available, by request, from the corresponding authors, due to matters of privacy.

## Abbreviations

ADC: Apparent diffusion coefficient
AUC: Area under the curve
CI: Confidence interval
DWI: Diffusion-weighted imaging
ICC: Interclass correlation coefficient
iCCA: Intrahepatic cholangiocarcinoma
MRE: Magnetic resonance elastography
OR: Odds ratio
ROIs: Regions of interest
