# Supplementary material for: Correlation analysis of MR elastography and Ki-67 expression in intrahepatic cholangiocarcinoma
Source: Insights Imaging. 2023 Nov 24;14:204. doi: 10.1186/s13244-023-01559-7 (PMC10673794; doi:10.1186/s13244-023-01559-7)
Supplement: Supplementary file 1 — Additional file 1: Table S1. Sequence parameters. Table S2. The intraclass correlation coefficient between two observers in the whole cohort for the qualitative MRI features. [file 13244_2023_1559_MOESM1_ESM.docx]

**Correlation analysis of MR elastography and Ki-67 expression in intrahepatic cholangiocarcinoma**

**ELECTRONIC SUPPLEMENTARY MATERIAL**

**Table S1.** Sequence parameters

| **Parameter** | **T1-weighted IP and OP imaging** | **Contrast enhanced T1-weighted imaging** | **T2-weighted imaging** | **Diffusion weighted imaging** |
| --- | --- | --- | --- | --- |
| Repetition time (ms) | 3.66 | 3.27 | 2000 | 2714 |
| Echo time (ms) | 1.2/2.4 | 1.45 | 106.2 | 63.3 |
| Field of view (mm^2^) | 400×300 | 400×300 | 380×380 | 380×300 |
| Matrix | 168×288 | 288×320 | 256×256 | 128×100 |
| Section thickness (mm) | 3 | 3 | 6 | 6 |
| Gap (mm) | 0 | 0 | 1.2 | 1.2 |
| Flip angle (degree) | 10 | 10 | 100 | 90 |
| Number of averages | 1 | 1 | 1 | 2 |

IP, in-phase; OP, opposed-phase

**Table S2.** **The intraclass correlation coefficient between two observers in the whole cohort for the qualitative MRI features**

| Parameters | Kappa (95%CI) |
| --- | --- |
| Tumor margin | 0.789(0.658-0.920) |
| Signal homogeneity | 0.920(0.831-1.000) |
| Arterial enhancement | 0.884(0.774-0.995) |
| Enhancement pattern | 0.839(0.687-0.992) |
| APHE | 0.863(0.748-0.979) |
| Capsule | 0.748(0.594-0.902) |
| Targetoid appearance | 0.804(0.664-0.943) |
| Bile duct dilation | 0.935(0.862-1.000) |
| Liver capsule retraction | 0.892(0.800-0.984) |
| Hemorrhage in mass | 0.852(0.566-1.000) |
| Necrotic or cystic portion | 0.889(0.794-0.984) |
| Central scar | 0.731(0.596-0.867) |
| Central darkness on T2WI | 0.855(0.751-0.958) |
| Vessel invasion | 0.913(0.817-1.000) |

APHE, arterial peritumoral hyperenhancement; T2WI, T2-weighted imaging
